# Anisotropic SpiralNet for 3D Shape Completion and Denoising

**DOI:** 10.3390/s22176457

**Published:** 2022-08-27

**Authors:** Seong Uk Kim, Jihyun Roh, Hyeonseung Im, Jongmin Kim

**Affiliations:** Department of Computer Science and Engineering, Interdisciplinary Graduate Program in Medical Bigdata Convergence, Kangwon National University, Chuncheon 24341, Korea

**Keywords:** shape denoising, shape completion, deep learning, graph convolutional networks

## Abstract

Three-dimensional mesh post-processing is an important task because low-precision hardware and a poor capture environment will inevitably lead to unordered point clouds with unwanted noise and holes that should be suitably corrected while preserving the original shapes and details. Although many 3D mesh data-processing approaches have been proposed over several decades, the resulting 3D mesh often has artifacts that must be removed and loses important original details that should otherwise be maintained. To address these issues, we propose a novel 3D mesh completion and denoising system with a deep learning framework that reconstructs a high-quality mesh structure from input mesh data with several holes and various types of noise. We build upon SpiralNet by using a variational deep autoencoder with anisotropic filters that apply different convolutional filters to each vertex of the 3D mesh. Experimental results show that the proposed method enhances the reconstruction quality and achieves better accuracy compared to previous neural network systems.

## 1. Introduction

Three-dimensional (3D) geometric data have consistently received much attention in the field of computer vision and graphics, and such data can be conveniently acquired by using various types of affordable 3D scanning [1] and depth camera devices. Most raw 3D shape data are represented as point clouds from low-cost hardware equipment. Three-dimensional mesh data structures that consist of numerous vertices, edges, and faces are also widely used in many industries and studies as well for the purposes of visualization, design, and manufacturing [2,3], as they are capable of efficiently storing 3D geometric shapes and reusing them. Converting 3D point cloud data into mesh data can be easily accomplished by making use of several commercial and public mesh libraries [4,5]. However, low-precision hardware and a poor capture environment will inevitably cause unordered point clouds with unwanted noise and holes that should be feasibly corrected while preserving the original shapes and details. Moreover, 3D mesh data converted from problematic point clouds are obviously not smooth and are unsuitable for use in isolation without additional post-processing steps such as 3D mesh completion and/or denoising.

The successful completion and refinement of a partial 3D mesh are known to be challenging and a crucial part of the modeling of high-quality industrial applications. Previous mesh filtering methods [6,7,8] mainly based on analytic approaches efficiently eliminate high-frequency noise in 3D mesh data and the geometric original details can simultaneously be retained. However, the parameters must be carefully chosen, and several instances of trial and error are mandatory before satisfactory results can be obtained. Moreover, new vertices that fully cover the partial meshes are seldom generated as they do not work very well when performing the shape completion task.

Recently, several studies have utilized convolutional neural networks (CNNs) to represent and process 3D mesh data. Convolutional mesh autoencoder (CoMA) [9] introduces spectral graph networks with Chebyshev filters to reduce the computational burden imposed by the high dimensional and large amount of training mesh data. However, transformation from the spatial to the spectral domain when training the networks results in a loss of the original shape of the mesh, thereby degrading the reconstruction accuracy. SpiralNet [10,11,12] outperforms CoMA owing to the well-designed spiral graph convolution operations. However, a spiral convolution filter with fixed coefficients is applied to the given mesh model and the representation power of the networks is therefore limited.

**Figure 1:**
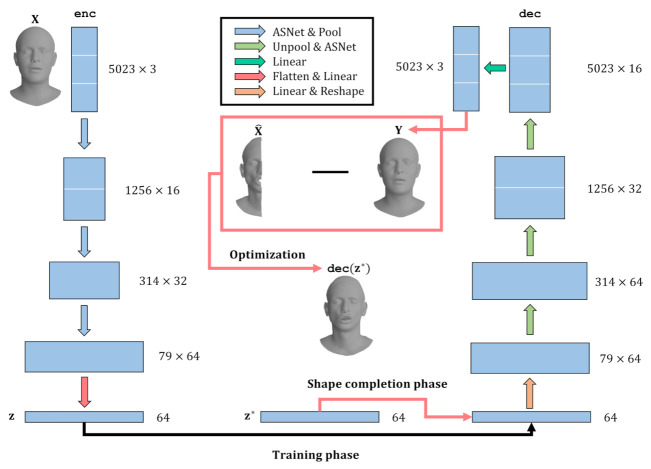
The architecture of the proposed model is based on anisotropic filters for 3D shape completion and denoising. In the prediction phase, we undertake partial shape completion, which iteratively optimizes the initial latent variable z by minimizing the errors between the valid parts of the input mesh and the corresponding predicted parts Y while holding the network parameters constant. The resulting output mesh $Y^{*}=\mathrm{dec}\left(z^{*}\right)$ is obtained by passing the optimal latent variable through a decoder.

**Figure 2:**
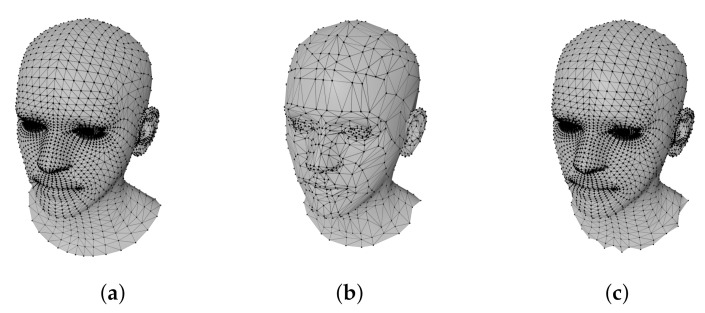
Mesh down- and upsampling results. The facial mesh (**a**) is downsampled for the pooling operation (**b**), then it is upsampled for the unpooling operation (**c**).

To overcome this issue, we propose a novel anisotropic graph convolution-based deep learning framework that performs 3D shape completion and refinement in a fully automatic manner (Figure 1). We utilize a graph convolutional autoencoder capable of extracting meaningful features from the mesh training data at each convolutional layer by locally observing the features to produce high-quality results. Specifically, we build the network upon SpiralNet to generate fine-grained and smooth meshes from the partial meshes with noise, and our anisotropic convolutions can apply a different filter to each vertex of the 3D mesh so as to improve the reconstruction power compared to previous CNN-based graph neural networks such as FeastNet, CoMA, and SpiralNets [9,10,11,12,13]. Inspired by the soft permutation of LSA-Conv [14], we model the spiral convolution weight matrix as a linear combination of the base matrices that are shared by all of the vertices of the 3D mesh. In contrast to conventional anisotropic filtering approaches that directly and independently apply different filters to each vertex, the method proposed here computes a small number of filter bases and corresponding coefficients for each vertex. Given that a typical 3D-scanned mesh has various types of noise and discontinuities, our anisotropic filtering approach is well-suited for this type of scanned mesh data. Our mesh autoencoder consists of several convolutional layers and up-and-down sampling based on quadratic mesh simplification for the pooling operations, as shown in Figure 1 and Figure 2. We find that incomplete 3D meshes passing through previous mesh autoencoders are not fully reconstructed and that holes remain. To cope with this problem, we run the optimization in the prediction phase. More precisely, we begin with the initial latent variables from the neural example to the decoder input, after which we update the latent variables iteratively to fill the holes fully.

In order to validate our system, we qualitatively and quantitatively compare the proposed method with those in previous work. In the experiments conducted in this study, our system achieves better performance as the anisotropic filters significantly improve the representation power and maintain the original shape while removing noise and filling the holes. We believe that our system can be considered as an efficient tool for the post-processing of scanned and captured 3D mesh data with randomly distributed noise and holes.

## 2. Related Work

Mesh restoration and modeling are important tasks in the computer vision and graphics field. Mesh-smoothing algorithms with the Poisson equation introduced earlier [15,16] are suitable for recovering small hole regions, but they do not work very well when handling larger ones. The linear 3D morphable model [17] learns 3D facial models obtained through scanning and expresses the texture and shape of the face in its partial space by using principal component analysis (PCA). SCAPE [18], one of the most well-known body models, utilizes PCA and runs a quadratic optimization process to represent natural human body postures. FLAME [19] can adjust facial expressions by using linear blend shapes with jaw and neck articulation. Most of these morphable models linearly represent the human body model, whereas the proposed approach effectively models the non-linearity of the human body structure by using well-designed deep learning frameworks.

Recently, deep-learning-based approaches have been attracting attention in the area of mesh data processing. For example, CNNs [20] have achieved great success in the image processing and computer vision fields [21]. CNNs are capable of learning the translation-invariant localized features of the training data. Recently, there have also been studies of various 3D mesh data structures [22,23,24,25,26,27]. Graph convolutional networks (GCNs) [28,29,30], popular deep learning frameworks for training and representing mesh data, analyze the geometric relationships between each node and its neighbors in a graph structure. There are two different types of graph convolutional architectures: the spectral and spatial types. Spectral GCNs [31] undertake convolutions by using the eigen-decomposition of the pre-defined graph Laplacian matrix, eventually converting all information pertaining to the vertices in the spatial domain into the spectral domain. Defferrad et al. [32] effectively reduced the computational costs incurred when training graph-structured data by utilizing the Chebyshev polynomial, which recursively approximates the graph Fourier transform without direct eigen-decomposition. CoMA [9] employs spectral GCNs to establish a 3D mesh autoencoder that also contains upsampling and downsampling layers based on a mesh simplification approach [33]. FeaStNet [13] performs its convolution operation in the spatial domain, and Litany et al. [34] proposes a CNN-based variational autoencoder (VAE) [35] for the probabilistic modeling of the latent space. Similar to our approach, it iteratively optimizes the latent variables by minimizing the errors between selected valid input mesh vertices and the predicted vertices while fixing the autoencoder parameters.

Spiral neural networks [10,11,12] have recently shown better results for processing and representing 3D mesh data when compared to earlier GCN-based methods. SpiralNet [10] defines the spiral structure that connects the vertices along the spiral trajectory and accurately finds the correspondences between two 3D meshes that have the same geometry but a different topology based on long short-term memory (LSTM) networks with the spiral structure. Instead of randomly selecting spiral vertices [10], Bouritsas et al. [11] proposes new ordered spiral sequences with a fixed length, resulting in improved performance for 3D mesh reconstruction with a fixed topology. SpiralNet++ [12] performs the convolution operation of concatenating the mesh vertices following a spiral trajectory that is fed to the multi-layer perceptrons (MLP) without truncation or zero-padding to construct the spiral structure [11]. Similar to previous dilated convolutions [36,37], dilated spiral convolution [12] has also been introduced to improve the performance without increasing the size of the spiral convolution. However, identical convolution filter weights are applied to all vertices in those SpiralNet-based methods, whereas we apply different filters efficiently and independently to each vertex. Experimental results show that the proposed method outperforms existing SpiralNet structures in terms of handling the holes and noise in the scanned 3D mesh. Also, the prediction speed of our mesh autoencoder is faster than CoMA, LSAConv, and SDConv [38], and similar to SpiralNet++.

## 3. Method

**Figure 3:**
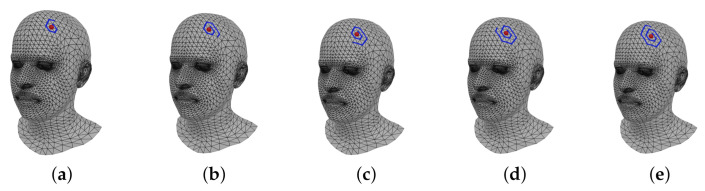
Visualization of spiral sequences with different user parameter. (**a**) l=9; (**b**) l=12; (**c**) l=15; (**d**) l=18; (**e**) l=21.

We propose a novel deep learning system that efficiently and automatically fills the holes and gaps of a partial 3D mesh shape with noise. We employ a deep variational autoencoder [35] that consists of an encoder and a decoder to predict the fine-grained 3D mesh structure. A latent space that embeds the meaningful spectral and spatial features of the 3D mesh data to achieve accurate hole filling and noise reduction outcomes is well established after training the proposed deep variational autoencoder. The input of our deep autoencoder are the complete 3D mesh vertices $X={\left[x_{0},x_{1},\ldots ,x_{N-1}\right]}^{⊤}\in R^{N\times F}$ and the output is $Y={\left[y_{0},y_{1},\ldots ,y_{N-1}\right]}^{⊤}\in R^{N\times F}$, where *F* is the feature dimension, F=3 represents the XYZ values of each vertex, and *N* denotes the total number of mesh vertices. Figure 1 shows our deep variational autoencoder in detail. The input 3D mesh X is encoded as z=enc(X) in the latent space and the latent vector $z\in R^{64}$ is decoded into the output 3D mesh Y=dec(z). In our system, there are six anisotropic spiral convolution layers; we add three up-and-down pooling layers and three linear layers to our neural network system, as shown in Figure 1. Specifically, SpiralNet++ [12] defines the *k*-ring and *k*-disk for each vertex *v* in the 3D mesh structure as follows:

(1) $$\begin{array}{cc} 0\mathrm{-ring}(v)= & \{v\},\\ k\mathrm{-disk}(v)= & \cup_{i=0,\ldots ,k}i\mathrm{-ring}\left(v\right),\\ (k+1)\mathrm{-ring}(v)= & N(k\mathrm{-ring}(v))/k\mathrm{-disk}(v), \end{array}$$

where 0-ring represents the starting vertex to define spiral sequences and *k*-disk denotes a union of *i*-rings, where i={0,…,k}. Here, N(V) denotes the set of neighboring indices of the vertices in the set V and (k+1)-ring is a set of vertex indices from N(V) excluding those included in *k*-disk. The spiral sequences S(v,l) for a single vertex index *v* are the following ordered set:

S(v,l)⊂(0-ring,1-ring,2-ring,…,k-ring),

where S(v,l) includes only a part of *k*-ring to define the length of the spiral sequences as the user-defined length *l*. Figure 3 shows the vertices that form the spiral according to the length *l*.

The spiral convolution input can be defined as $x_{i}={\|}_{j\in S(v_{i},l)}x_{j}$, where the *l* vertex features included in the spiral sequences $S(v_{i},l)$ of the starting vertex index $v_{i}$ are concatenated into a vector $x_{i}$ of the *i*-th vertex. The convolutional output $y_{i}$ is then computed with the vertex feature $x_{i}\in R^{F_{in}\cdot l}$, convolution weight matrix $W\in R^{F_{out}\times F_{in}\cdot l}$, and bias vector $b\in R^{F_{out}}$ according to Equation (2):

(2) $$y_{i}=Wx_{i}+b$$.

LSA-Conv [14] constructs the vertex features $X_{i}={\left[x_{i,0},x_{i,1},\ldots ,x_{i,K-1}\right]}^{⊤}\in R^{K\times F_{in}}$ for each vertex and the corresponding local 1-ring neighbors, where K−1 is the number of neighbors for each convolutional layer. In addition, a trainable weight matrix $P_{i}\in R^{K\times K}$ is plugged into Equation (2) in a soft permutation form to sort the 1-ring neighbors for each vertex on the mesh. Therefore, the output $y_{i}$ is obtained by the equation below:

(3) $$y_{i}=W\left[\mathrm{vec}\left(f\left(P_{i}X_{i}\right)\right)\right]+b,$$

where vec converts the vertex feature matrix X into a column vector and *f* represents the non-linear activation function.

**Figure 4:**
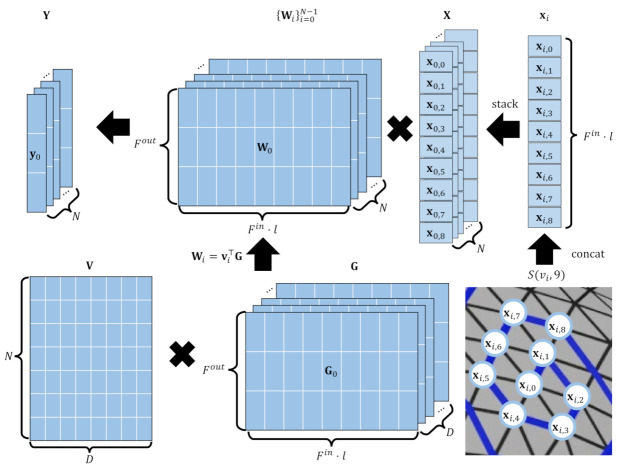
In the proposed anisotropic spiral convolution method, the spiral sequences $S(v_{i},l=9)$ corresponding to the *i*-th vertex feature are concatenated from $x_{i,0},x_{i,1},\ldots ,x_{i,8}$; this is denoted as $x_{i}$. We linearly parameterize the convolutional filter weights ${\left\{W_{i}\right\}}_{i=0}^{N-1}$ into the base matrices $G\in R^{D\times F_{out}\times F_{in}\cdot l}$ and their coefficients $V\in R^{N\times D}$ to ensure that each vertex has a different and desired convolutional filter. In doing so, our method enhances the representation power compared to the conventional isotropic convolutional filters.

LSA-Conv [14] devised the idea of effectively sorting all vertices by approximating a set of weight matrices ${\left\{P_{i}\right\}}_{i=0}^{N-1}\in R^{N\times K\times K}$ as a linear combination of trainable basis matrices $V\in R^{N\times D}$, where the number of bases is *D*, which is significantly smaller than the number of vertices, *N*. Weight matrix parameterization enables the automatic sorting of each vertex so that graph convolution can perform well. We modify this approach to enhance the network representation power by using anisotropic spiral convolutional filters such that each vertex has a different and desired convolution filter. Instead of directly and independently applying different filters to each vertex, our subspace design computes a small number of filter bases and corresponding coefficients for each vertex. We linearly parameterize the convolution weights of SpiralNet++ [12] as follows:

(4) $$\begin{array}{cc} W_{i}= & v_{i}^{⊤}G\\ b_{i}= & v_{i}^{⊤}C\\ y_{i}= & W_{i}x_{i}+b_{i}, \end{array}$$

where ${\left\{W_{i}\right\}}_{i=0}^{N-1}\in R^{N\times F_{out}\times F_{in}\cdot l}$ includes spiral convolutional filters of length *l* for *N* vertices, as represented in $G\in R^{D\times F_{out}\times F_{in}\cdot l}$ and its corresponding coefficient matrix $V={\left[v_{0},v_{1},\ldots ,v_{N-1}\right]}^{⊤}\in R^{N\times D}$, which includes *D*-dimensional vectors. Here, $B={\left[b_{0},b_{1},\ldots ,b_{N-1}\right]}^{⊤}\in R^{N\times F_{out}}$ denotes the bias matrix, and each bias vector is also linearly parameterized with a vector of bases $C\in R^{D\times F_{out}}$. The computational cost of our approach is similar to that in [12] while the reconstruction accuracy is greatly improved when compared to other neural network architectures, as ours allows locally anisotropic convolution filtering for all vertices on the 3D mesh. Figure 4 shows how our anisotropic spiral filter works.

The proposed deep variational autoencoder trains the encoder to build a variational distribution q(z|X). The decoder is then trained to generate the resulting 3D mesh Y from the latent vector z. We train the neural network with the KL divergence and reconstruction $L_{2}$ losses. We use the normal distribution N(0,I) to make the variational distribution q(z|X) sample similarly as the prior distribution p(z) in the latent space. The loss function of our network to be minimized is φ(X,Y)−λKL(q(z|X)‖p(z)), where φ is the Euclidean distance between X and Y with the $L_{2}$ norm and λ is a constant weight value for KL divergence. In our experiments, we set λ to $10^{-8}$.

**Figure 5:**
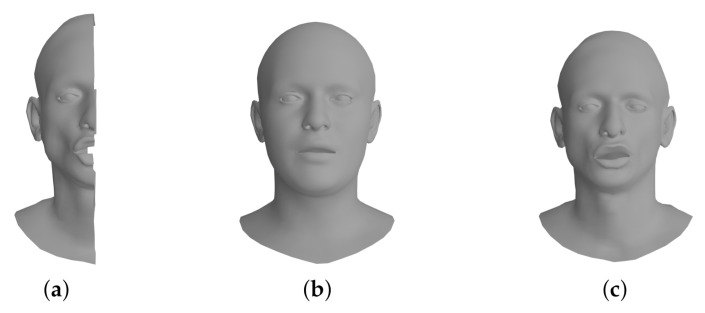
The leftmost mesh shows the partial input mesh (**a**). The initial mesh is obtained by the decoder before performing network optimization (**b**), and the rightmost mesh shows the result obtained from the decoder after the iterative optimization step (**c**).

In the prediction phase, we iteratively optimize the decoder parameters only to reconstruct the incomplete 3D mesh faithfully. First, we generate the initial facial mesh to be optimized from the latent vector z that follows a normal distribution N(0,I) (see Figure 5b). Second, we pass the initial latent vector z into the decoder and the latent vector is optimized by iteratively solving $\|\hat{X}-\Pi \left(\mathrm{dec}\left(z\right)\right){\|}_{2}^{2}$, where $\hat{X}$ is the incomplete input mesh (see Figure 5a) and Π is a selection matrix. Hence, the resulting output mesh $Y^{*}=\mathrm{dec}\left(z^{*}\right)$ is obtained from the optimal latent vector $z^{*}$ (see Figure 5c). We would like to refer the reader to [34] for the optimization of partial shape completion in detail.

## 4. Results

We quantitatively and qualitatively compared the proposed method with previous network systems such as CoMA, SpiralNet++, LSAConv, and SDConv, for the shape reconstruction and completion of the 3D mesh. We used two different datasets to train our neural networks. First, the MPI Dynamic FAUST dataset [39] includes full-body character motion. Our network was trained by using 37,557 frames out of a total of nearly 40,000 frames with a total of 10 actors and approximately 13 expressions, and was evaluated by using 3563 frames that were not used for training. The second dataset is CoMA, which includes various types of facial meshes of 18,845 frames with a total of 12 people and 12 expressions; our network was also evaluated for 1620 frames that were not included in the training dataset. All experiments were conducted by using PyTorch [40] on a system with an AMD Ryzen 9 5900X processor, 64 GB of memory, and an NVIDIA RTX 3080Ti GPU. The specific structures of the encoder and decoder are shown in Table 1. All networks were trained by using the Adam optimizer [41], and the learning rate of 0.001 and 100 epochs were utilized. For LSAConv and SDConv, 300 epochs were used.

**Figure 6:**
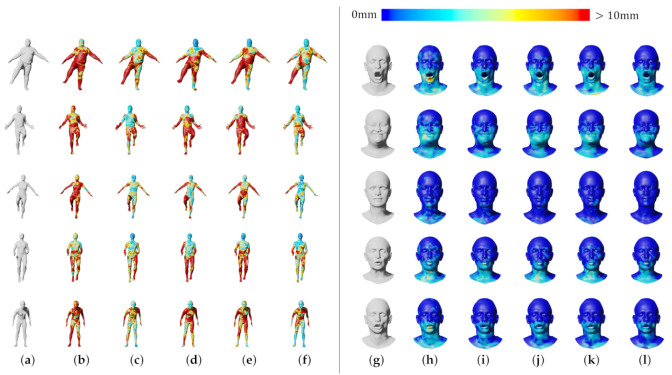
Qualitative comparison of reconstruction errors. (**a**–**f**): DFAUST dataset, (**g**–**l**): CoMA dataset. (**a**,**g**): Ground truth; (**b**,**h**): CoMA; (**c**,**i**): SpiralNet++; (**d**,**j**): LSAConv; (**e**,**k**): SDConv; (**f**,**l**): Ours.

**Figure 7:**
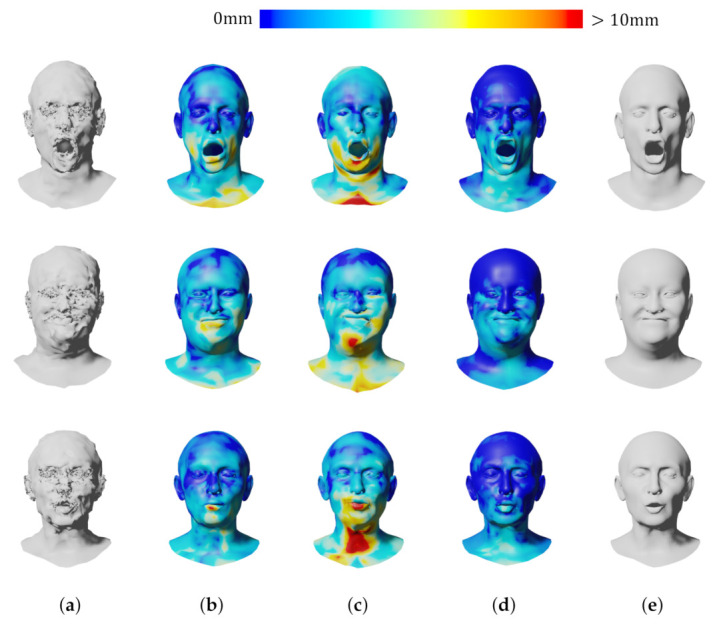
Qualitative comparison of shape denoising. (**a**) Input; (**b**) CoMA; (**c**) SpiralNet++; (**d**) Ours; (**e**) Ground truth.

**Figure 8:**
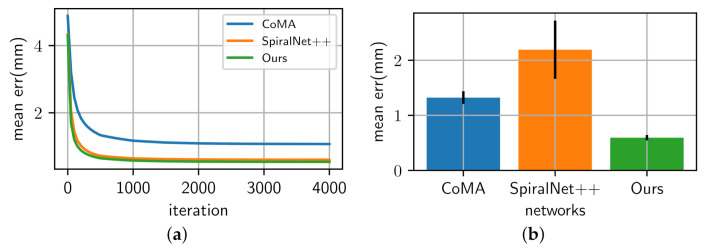
(**a**) This figure shows that our neural network system accomplishes faster convergence of the network optimization with fewer errors for the shape completion when compared with other neural network systems. (**b**) Our system achieves lower variance and errors than other neural network systems for the shape denoising task. All the errors are measured in millimeters.

Table 2 and Figure 6 show the reconstruction errors compared to the other neural networks. The proposed network outperforms CoMA, SpiralNet++, and SDConv on 3D mesh reconstruction. Although our neural network has a bit more parameters than SpiralNet++, the prediction speed is similar to that of SpiralNet++ when performing the mesh reconstruction as our anisotropic convolution computation in Equation (4) is exactly same as the spiral one in Equation (2) other than computing with $\left\{W_{i},b_{i}\right\}$. The precomputed $\left\{W_{i},b_{i}\right\}$ during the training step are reused in the prediction step, and we could not observe a meaningful prediction speed difference between ours and SpiralNet++. In addition, we synthetically and randomly added Gaussian noises on the input mesh data to test how well our system gets rid of the noises in the mesh data. In Figure 7, SpiralNet++ (Figure 7c) and CoMA (Figure 7b) successfully removed the given Gaussian noises, and CoMA showed better results than SpiralNet++. Our system achieved the smallest errors in every case that we have tested while preserving the original details of the mesh data (Figure 7d and Figure 8).

**Figure 9:**
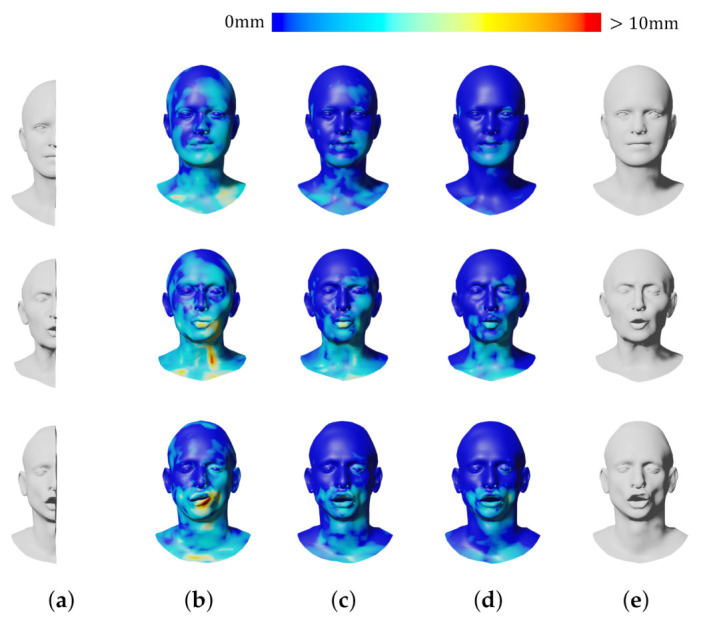
Qualitative comparison of shape completion. (**a**) Input; (**b**) CoMA; (**c**) SpiralNet++; (**d**) Ours; (**e**) Ground truth.

We also compared our approach with other networks for the 3D shape completion task and removed half of the vertices on the input mesh for the test. During the prediction step, the latent vector z was optimized 4000 times. We measured the average errors in each iteration (see Figure 8) and the average errors of the resulting mesh obtained at the last iteration (see Table 3). Figure 9 shows that CoMA (Figure 9b) generated a plausible overall shape of the mesh but did not produce expression details well. Meanwhile, SpiralNet++ (Figure 9c) showed better performance in terms of both overall shape completion and expressions, similar to the ground truth. However, some unnatural results were produced in the region of the lips that did not exist in the input mesh data. Our method (Figure 9d) successfully filled the holes and reconstructed the overall mesh shapes well while fewer error results occurred in missing regions of input mesh data. Table 3 presents comparisons of the 3D shape completion errors for each different mesh, where we find that the proposed method resulted in a smaller average error value compared to the other neural networks.

## 5. Conclusions

We have presented a novel anisotropic spiral neural network that faithfully reconstructs and completes a partial 3D mesh. We found that the proposed method could enhance the representation power owing to applying different convolutional filters to each vertex on 3D mesh. We show that our anisotropic filter can improve the reconstruction and shape completion accuracy for a facial and body mesh model. Our system can be useful for post-processing to obtain visually appealing mesh results without noticeable artifacts.

There will be several desirable future works. It would be interesting to extend our work to handle complex and arbitrary mesh structures mainly used in various engineering fields. We can apply the anisotropic spiral filters to other different types of data such as images and video sequences. Specifically, ours offers the possibility of successfully reconstructing the defective and noisy medical image data obtained from magnetic resonance imaging (MRI), computed tomography (CT), and ultrasonography [42]. Therefore, we believe that detecting abnormal tissues based on the post-processed medical image data from our model can be helpful to treat the patients. From the promising results in our work, we plan to establish a modified system that can handle highly dense mesh data as well as extremely problematic mesh data, in which it is difficult to differentiate original features from noise. To do so, we will incorporate attention mechanisms in our neural networks to achieve further improvement of the shape completion and denoise because they have the ability to selectively focus on relevant features during the training procedure.

## Figures and Tables

**Table 1:** The architectures of our network and other neural networks tested in this paper (CoMA and SpiralNet++) are shown in detail. Note that ASNet represents our anisotropic convolutional filter shown in Figure 4 and that the input and output shapes of each layer are identical across all networks. * For variational autoencoder scheme, it requires two linear layers for mean ($\mu \in R^{64}$) and standard deviation ($\sigma \in R^{64})$.

CoMA / SpiralNet++ (Encoder)			Ours (Encoder)		
**Layer**	**Input**	**Output**	**Layer**	**Input**	**Output**
CoMA/SpiralNet	5023×3	5023×16	ASNet	5023×3	5023×16
Pool	5023×16	1256×16	Pool	5023×16	1256×16
CoMA/SpiralNet	1256×16	1256×32	ASNet	1256×16	1256×32
Pool	1256×32	314×32	Pool	1256×32	314×32
CoMA/SpiralNet	314×32	314×64	ASNet	314×32	314×64
Pool	314×64	79×64	Pool	314×64	79×64
Flatten	79×64	1×5056	Flatten	79×64	1×5056
Linear	1×5056	1×64 *	Linear	1×5056	1×64 *
**CoMA / SpiralNet++ (Decoder)**			**Ours (Decoder)**		
**Layer**	**Input**	**Output**	**Layer**	**Input**	**Output**
Linear	1×64	1×5056	Linear	1×64	1×5056
Reshape	1×5056	79×64	Reshape	1×5056	79×64
Pool	79×64	314×64	Pool	79×64	314×64
CoMA/SpiralNet	314×64	314×64	ASNet	314×64	314×64
Pool	314×64	1256×64	Pool	314×64	1256×64
CoMA/SpiralNet	1256×64	1256×32	ASNet	1256×64	1256×32
Pool	1256×32	5023×32	Pool	1256×32	5023×32
CoMA/SpiralNet	5023×32	5023×16	ASNet	5023×32	5023×16
CoMA/SpiralNet	5023×16	5023×3	Linear	5023×16	5023×3

**Table 2:** Comparisons of the reconstruction errors, number of network parameters, and speed for CoMA, SpiralNet++, LSAConv, SDConv, and ours. All the reconstruction average errors are measured in millimeters.

Network	Dataset					
	DFAUST			CoMA		
	Error	Params	Frame/sec	Error	Params	Frame/sec
CoMA	12.416	1390K	548.0±5.4	0.6661	1031K	450.6±8.6
SpiralNet++	7.510	1418K	1482.9±24.9	0.4236	1059K	894.4±8.7
LSAConv ^1^	10.493	2540K	347.2±3.4	0.4203	1723K	356.0±3.0
SDConv ^1^	10.488	**564K**	494.8±2.4	0.4525	**443K**	448.5±5.1
Ours	**6.151**	2147K	1463.1±24.3	**0.3551**	1750K	912.3±8.4

^1^ Zhongpai Gao, https://github.com/Gaozhongpai/SDConvMesh. (accessed on 14 July 2022).

**Table 3:** Quantitative comparison of shape completion by using different actor models from the CoMA dataset. All the average errors are measured in millimeter (mm).

Network	Actor ID												Mean
	0137	3272	0024	0138	3274	3275	0128	3276	3277	3278	3279	0223	
CoMA	1.073	1.179	1.057	1.107	1.161	0.890	1.306	1.059	0.999	0.926	1.040	0.873	1.071
SpiralNet++	0.489	**0.568**	0.592	0.675	0.699	0.460	0.760	0.594	0.558	0.516	0.611	0.473	0.603
Ours	**0.447**	0.574	**0.495**	**0.593**	**0.625**	**0.389**	**0.759**	**0.533**	**0.474**	**0.437**	**0.530**	**0.419**	**0.541**

## Data Availability

Not applicable.

## Abbreviations

The following abbreviations are used in this manuscript:

3D	Three-dimensional
CNN	Convolutional neural network
CoMA	Convolutional mesh autoencoder
PCA	Principal component analysis
GCN	Graph convolutional network
LSTM	Long short-term memory
MLP	Multi-layer perceptron
MRI	Magnetic Resonance Imaging
CT	Computed Tomography
